# Measurement error in total energy intake in the United Kingdom National Diet and Nutrition Survey (2008–2015)

**DOI:** 10.1016/j.ajcnut.2025.09.037

**Published:** 2025-09-24

**Authors:** Michelle C Venables, Caireen Roberts, Dan Griffiths, Elise R Orford, Dave Collins, Albert Koulman, Nicholas J Wareham, Polly Page

**Affiliations:** Medical Research Council Epidemiology Unit, University of Cambridge, Cambridge, United Kingdom

**Keywords:** energy intake, measurement error, free-living energy expenditure, stable isotopes, diet diary

## Abstract

**Background:**

Measurement error in the estimation of energy intake (EI) from self-reported dietary assessment is a common phenomenon, which can lead to implications for epidemiological studies and surveys that aim to determine relationships between diet and health.

**Objectives:**

The aim of this study was to examine measurement error in estimated EIs within the UK National Diet and Nutrition Survey (NDNS, 2008–2015), to determine the degree of error, if measurement error has changed over the course of data collection periods, and to identify any related associations.

**Methods:**

Data from 770 (383 males and 387 females) participants from the UK NDNS recruited between 2008 and 2011 and 2013 and 2015 who had height and weight measurements, ≥3 of 4 diet diary days, and total energy expenditure estimations using the doubly labeled water (DLW) method were used for this secondary analysis study. Data were obtained via the UK Data Service (https://ukdataservice.ac.uk/).

**Results:**

Overall, when compared with DLW total energy expenditure measurements, EI from self-report dietary data was underestimated by 27% [95% confidence interval (CI): 25, 28%], and did not differ significantly in combined age/sex strata between survey years 2008–2011 and survey years 2013–2015 (25%; 95% CI: 23, 27% compared with 28%; 95% CI: 26, 31%, respectively). Likewise, measurement error in reported EI in the majority of age/sex strata generally remained stable. Measurement error in reported EI was associated with body mass index (−0.02 ratio units per kg/m^2^; 95% CI: −0.02, −0.01 ratio units per kg/m^2^), mean difference (56 kcal/kg/m^2^; 95% CI: 47.1, 64.3 kcal/kg/m^2^), age (mean difference: −8.4 kcal/y; 95% CI: −1.2, −4.8 kcal/y), and sex (mean difference in females: −173 kcal; 95% CI: −252.7, −93.9 kcal) and not associated with ethnicity, education, equivalized income or geography.

**Conclusions:**

We show that although measurement error in estimation of EI is evident within the UK NDNS, it has remained similar. Measurement error was associated with body mass index, age, and sex.

## Introduction

The National Diet and Nutrition Survey (NDNS) provides essential evidence on the diet and nutrition of the United Kingdom population. The accurate measurement of food and drink intake is a major challenge in dietary surveillance and epidemiological studies. Such research relies on feasible and pragmatic approaches, commonly self-reported measures, which are prone to measurement error. Measurement error from self-reported diet intake is a complex multifactorial issue, related to the complexity of assessment, conscious and unconscious causes, and methodological factors. Error in the estimation of energy intake (EI; derived from participant self-reports of food and drink consumption) can be quantified, and it has been shown that EI is typically underestimated in population dietary datasets [[1], [2], [3]]. Given the reliance on self-reported measures of food intake, even with careful attention to research methods across the dietary data collection and processing cycle, it is not possible to entirely prevent or account for measurement error. Efforts to quantify measurement error in studies help to reveal some of the extent and nature of error and bias in self-report dietary data, and inclusion of such measures in ongoing programs such as the NDNS offers a way to track aspects of measurement error over time.

A simple means by which to appreciate measurement error in estimates of EI derived from reported dietary intake is to calculate the minimum EI required to maintain current weight, using basal metabolic rate (BMR) and physical activity level (PAL) [4,5]. The difference between estimated intake and the minimum required intake shows the likely measurement error. Cutoffs can be applied to estimated EI data to determine the plausibility of self-reporting, as provided by Goldberg et al. [6] (Goldberg cutoffs) and later developed by Black [7,8].

EI can also be measured objectively by proxy, by estimating energy expenditure using doubly labeled water (DLW). The DLW method is widely regarded as the reference method for measuring total energy expenditure (TEE) in free-living individuals [9]. The method estimates water turnover and carbon dioxide production in individuals after a dose of the stable isotopes of hydrogen (^2^H) and oxygen (^18^O) over a period of measurement (7–14 d). Provided an individual is in energy balance, TEE is equal to EI; the use of DLW can therefore provide an index of measurement error in estimated EI derived from self-reported dietary data.

Because of high cost, burden, and logistic complexities, the DLW method is not routinely used in research, and the NDNS is one of the few large-scale nutrition surveys to have regularly incorporated DLW as an objective biomarker for the assessment of TEE. Although DLW data are collected from only a subsample of NDNS participants, its relatively large scale provides unique insight and direct quantification of measurement error in estimated EIs in a national survey context. Here, we present this assessment and monitoring, providing the magnitude of measurement error of EI estimated from self-reported diet in the UK NDNS (2008–2015) by comparison with TEE estimated by DLW, and a comparison between study time points.

## Methods

The NDNS has been running since 2008 and is a continuous, cross-sectional survey designed to assess nutrient intake and nutritional status of the general population aged ≥1.5 y, living in households in the United Kingdom. Complete methodology for the NDNS for 2008–2019 are described elsewhere [10]. In summary, the survey used a stratified sampling design to generate a random sample of UK households each year from which, during this period, a nationally representative core sample of ∼1000 participants per year (500 children aged 1.5–18 y and 500 adults aged ≥19 y) were recruited using an in-home interviewer-led field model (in some years, the sample was boosted to increase numbers in Scotland, Wales, and Northern Ireland). For this period, DLW was included in each 4- to 5-y commissioning phase and was collected in a subset (∼10%) of participants in survey years 2008–2011 [11] and 2013–2015 [12].

All data for this secondary analysis of the NDNS DLW substudies were obtained through the UK data service (https://ukdataservice.ac.uk/) [13,14].

### Participants

The present analysis included data from 770 participants (383 males, 387 females) who completed the NDNS DLW substudies either in survey years 2008–2011 or survey years 2013–2015 (a participant flow chart is provided in Supplemental Figure 1), including dietary assessment using a 4-d unweighed paper food diary and a 10-d estimation of TEE using DLW. Recruitment to the DLW substudy was a convenience sample, with recruitment into prespecified age/sex strata from age ≥4 y (4–10, 11–15, 16–49, 50–64 and ≥65 y), subject to completion of 3 of the 4 diet diary days, until the age/sex quotas were filled. In survey years 2008–2011, age/sex recruitment targets were set at *n* = 40, whereas in survey years 2013–2015, a larger number was recruited in the middle age strata to account for a larger observed variability in these strata; resulting in quotas of 4 to 10 y (*n* = 60), 11 to 15 y (*n* = 80), 16 to 49 y (*n* = 100), 50 to 64 y (*n* = 80), and ≥65 y (*n* = 60), with equal numbers within groups for each sex. These targets were selected as this enabled detection of an EI:TEE ratio of 0.88 within a substudy and a change in EI:TEE ratio between substudies of 0.12 within each age/sex strata as statistically significant at the 5% level with 80% power. Dosing for the DLW measurement was carried out by NDNS interviewers in participants’ homes with the aim to give the dose within 2 wk after the dietary recording period had completed. This resulted in a mean of 9 d (minimum 0 d, maximum 40 d) between the last diary day and DLW dosing.

The NDNS conforms to the Declaration of Helsinki and operates under United Kingdom National Health Service Health Research Authority Research Ethics Committee approval (Years 1–5, Oxfordshire REC A, REF 07/H0604/113; Years 6–10 and 11–15, East of England-Cambridgeshire South REC, REF 13/EE/0016). All participants aged ≥16 y provided written informed consent, and participants between the ages of 4 and 16 y provided assent with written informed consent provided from their legal guardian for participation in the DLW substudies.

### Non-dietary variables

Anthropometric measurements were taken by the NDNS interviewer in the participants’ home. Height and weight were measured, using a portable stadiometer and weighing scales, measuring to the nearest 0.1 cm and 0.1 kg, respectively. Body mass index (BMI) (in kilograms per meters squared) was calculated from these measures. Income derived by a standard methodology for equivalization (equivalized income) that adjusts household income to account for different demands on resources by considering the household size and composition [15] and education level (highest qualification gained) were used as measures of socioeconomic status. Data on ethnicity, smoking, and drinking status were also collected. The United Kingdom geographical regions were categorized as Government Office Regions as follows: North East, North West, Yorkshire and the Humber, East Midlands, West Midlands, the East of England, South West, London, South East, Scotland, Wales, and Northern Ireland.

### Total EI and other dietary variables

Dietary intake was collected over 4 consecutive days using a paper food diary with estimated portion weights (participants were not asked to weigh their foods). Participants were asked to self-record all food and drinks consumed both at home and away from home, including at school, work, and leisure activities. Children aged ≥12 y were encouraged to complete the diaries themselves, whereas for children below this age, the parent/carer was asked to complete the diary with the child’s input as appropriate and to include meals consumed while the child was at school. Participants were asked to describe foods and drinks in as much detail as possible and to record how much they had consumed. Food portion photographs were provided for a small number of frequently consumed foods to guide portion estimation, but the majority of portion sizes were recorded by participants in household measures (e.g., 2 dessert spoons of baked beans, Kit Kat—2 fingers) or, for packaged foods, the weight indicated on the packet. All participants were asked to keep and provide any packaging with their completed diaries to aid food and portion size coding. Portion descriptions in the diary were later converted into portion weights by the research team. Diaries were collected and reviewed by the NDNS interviewer in the participants’ home for any missing or ambiguous entries and were subsequently coded by trained staff using a bespoke diet coding and analysis program (Diet In, Nutrients Out [DINO]) [16] to calculate nutrient intake. Coded foods and dietary supplements were linked to food composition data from the NDNS nutrient database [17], which is compiled with information from the United Kingdom Composition of Foods Integrated Dataset [18], the Food Standards Agency (FSA) Standard Recipes Database [19], and manufacturers’ data gathered through food labels and web information. Portion sizes were assigned using information from food diary entries and data from manufacturer or retailer websites. A similar approach was used for coding items reported as consumed from fast food and similar outlets. Research staff referred to the FSA Food Portion Sizes book [20] to provide standard weights for unprocessed foods such as fruit and vegetables and as a reference for manufactured items when no other data were available. This source also provided weights for default small, medium, and large portions of typical dishes for adults. For children, age-appropriate portions were used based on the analysis of portion sizes consumed in previous NDNS, based on weighed records [21]. For foods consumed at school, portion sizes and nutrient content were informed by data collected from school caterers and school meal surveys [22]. Portion sizes were also obtained from packaging supplied by participants or by undertaking specific projects to update portion size estimates. Food and nutrient intakes were calculated within DINO from which estimates of average total EI and macronutrient composition of the diet (fat, carbohydrate, protein, and alcohol) were determined [17] alongside other dietary analyses.

### Total energy expenditure

For DLW, participants provided a predose baseline urine sample to establish the natural abundance of the stable isotopes, drank a bodyweight-specific dose of DLW (80 mg/kg body mass of ^2^H_2_O and 150 mg/kg body mass of H_2_^18^O) followed by collection of spot urine samples on each of the subsequent 10 d. Carbon dioxide production rate was calculated using the multipoint method of Schoeller et al. [9]. A calculated food quotient from the dietary assessment was determined using the calculation of Jequier et al. [23] and converted to TEE using the energy equivalent of carbon dioxide from Elia and Livesey [24].

Urine samples were analyzed, in duplicate, for ^18^O enrichment using the carbon dioxide equilibration method of Roether [25]. Any participants with <6 usable post dose samples were excluded. Briefly, 0.5 mL of sample was transferred into 12-mL vials (Labco Ltd), flush-filled with 5% carbon dioxide in nitrogen gas and equilibrated overnight while agitated on rotators (Stuart, Bibby Scientific). Headspace of the samples were then analyzed using a continuous flow isotope ratio mass spectrometer (IRMS) (AP2003, Analytical Precision Ltd). For ^2^H enrichment, 0.4 mL of sample was flush-filled with hydrogen gas and equilibrated over 6 h in the presence of a platinum catalyst. Headspace of the samples were then analyzed using a dual-inlet IRMS (Isoprime, GV Instruments) for survey years 2008–2011 and continuous flow IRMS for survey years 2013–2014 (Sercon ABCA-Hydra 20-22, Sercon Ltd).

To compare the 2 analytical systems, we measured the isotopes in urine samples of 2 individuals 10 times across 10 wk on both systems and calculated TEE. For survey years 2008–2011 (measured with the AP2003 for oxygen and the Isoprime for hydrogen), the coefficient of variation (CV) was 1.97% and for survey years 2013–2015 (measured with the AP2003 for oxygen and the Hydra for hydrogen), the CV was 4.16%. There was no evidence that the change in the analytical system introduced any bias.

All samples were measured alongside secondary reference standards previously calibrated against the primary international standards Vienna-Standard Mean Ocean Water (vSMOW) and Vienna-Standard Light Antarctic Precipitate (International Atomic Energy Agency, IAEA). All enrichment data are expressed in per mil (‰) with respect to vSMOW on the delta scale.

For age, sex, weight, and height (see section on nondietary variables), the BMR was estimated using the calculation of Schofield et al. [4], and PAL was determined by dividing TEE by BMR. Physical activity energy expenditure was calculated as TEE minus BMR minus diet-induced thermogenesis (constant of 10% of TEE).

### Estimated measurement error for EI

Measurement error in the estimation of EI within the UK NDNS dietary data was determined using TEE measured by DLW, compared with estimates of total EI derived from analysis of foods and drinks self-reported by participants in the unweighed 4-d food diary. The data are reported as a ratio (EI:TEE) and a mean difference (TEE-EI). For the following categories and covariates, we determined the association with the measurement error of EI: age (in years); sex (male, female); Government Office Region (North East, North West, Yorkshire and the Humber, East Midlands, West Midlands, East of England, London, South East, South West, Wales, Scotland, Northern Ireland); ethnic group (White, Mixed, Black, Asian, Other); income (£); BMI (in kilograms per meters squared); education (≥16 y) (Degree or equivalent; Higher education, below degree; A-levels or equivalent; General certificate of secondary education; Foreign or other; No qualifications; Still in full-time education); smoking status (nonsmoker, smoker); and frequency of drinking alcohol (≥8 y) (≥5 d/wk, 3 or 4 d/wk, Once or twice a week, Once or twice a month, Once every couple of months, Once or twice a year, never in the last 12 months).

### Statistics

Participant characteristics were presented as mean (95% confidence interval [CI]) for continuous variables and as *n* for categorical variables. Although survey weights are available for the main NDNS survey data variables, no weights are available for the DLW substudies, so all analyses are unweighted.

Associations between survey years, age, sex, government office region, ethnic group, income, BMI, education, smoking status, and drinking frequency compared with absolute magnitude of measurement error (TEE-EI) and ratio of measurement error (EI:TEE) were calculated through the use of multiple linear regression models.

The distribution of all variables was investigated and tested for normality, and no transformation was necessary. *t* tests were performed to determine differences in EI, TEE, and EI:TEE between study periods for each age/sex strata, and a 2-tailed *P* value < 0.05 (without multiple comparison correction) was considered to be statistically significant in all analyses. R software (version 3.6.1) was used for all statistical analyses.

## Results

Characteristics of the 770 study participants (371 and 399 for survey years 2008–2011 and survey years 2013–2015, respectively) are shown in Table 1, with the TEE (measured using DLW) and total EI (estimated from dietary assessment) presented for each age/sex strata and with both sexes combined per age strata. For all groups, there is a breakdown of the TEE into the BMR (calculated using Schofield method) and the estimated physical activity. For EI, we provide the percentages of macronutrient contribution to the EI, as well as that of alcohol, determined from the coded dietary data. The EI and TEE for each age/sex strata are displayed separately for the 2 periods (2008–2011 and 2013–2015) in Table 2.

**TABLE 1:** Characteristics of the United Kingdom National Diet and Nutrition Survey doubly labeled water participants (2008–2015)^1^.

	Females					Males					Total				
Age group	4–10	11–15	16–49	50–64	65+	4–10	11–15	16–49	50–64	65+	4–10	11–15	16–49	50–64	65+
Age (y)	7.5	13.4	31.9	57.0	72.9	7.1	12.8	29.2	56.4	73.3	7.3	13.1	30.6	56.7	73.1
	(7.0, 7.9)	(13.1, 13.7)	(29.6, 34.2)	(56.0, 58.0)	(71.3, 74.4)	(6.7, 7.5)	(12.5, 13.1)	(26.9, 31.6)	(55.4, 57.4)	(71.8, 74.9)	(7.0, 7.6)	(12.9, 13.3)	(29.0, 32.2)	(56.0, 57.4)	(72.0, 74.2)
*n*	73	80	91	79	64	74	76	89	83	61	147	156	180	162	125
Survey year (*n*)															
2008–2011	41	38	40	37	32	41	34	38	41	29	82	72	78	78	61
2013–2015	32	42	51	42	32	33	42	51	42	32	65	84	102	84	64
Anthropometry															
Height (cm)	127	159	164	162	160	126	159	178	175	172	126	159	171	169	166
	(124, 130)	(158, 161)	(162, 165)	(160, 163)	(158, 162)	(123, 128)	(157, 162)	(176, 179)	(174, 177)	(170, 173)	(125, 128)	(158, 161)	(169, 172)	(167, 170)	(164, 167)
Weight (kg)	28.5	54.7	70.0	76.5	73.5	26.6	53.0	82.7	86.7	82.8	27.5	53.9	76.3	81.7	78.1
	(26.4, 30.6)	(51.8, 57.6)	(66.5, 73.5)	(73.0, 80.0)	(69.9, 77.1)	(25.1, 28.1)	(49.9, 56.1)	(78.7, 86.7)	(83.4, 90.0)	(79.3, 86.4)	(26.2, 28.8)	(51.8, 56.0)	(73.5, 79.1)	(79.2, 84.2)	(75.4, 80.7)
BMI (kg/m^2^)	17.2	21.4	26.2	29.3	28.7	16.6	20.6	26.2	28.2	28.0	16.9	21.0	26.2	28.7	28.3
	(16.5, 17.8)	(20.4, 22.3)	(24.9, 27.5)	(28.0, 30.6)	(27.4, 30.0)	(16.1, 17.1)	(19.7, 21.5)	(25.0, 27.5)	(27.2, 29.2)	(27.0, 29.0)	(16.5, 17.3)	(2.4, 21.6)	(25.3, 27.1)	(27.9, 29.6)	(27.5, 29.2)
Body fat (%)	26.2	31.5	37.0	43.0	43.4	20.8	25.7	27.1	31.5	34.2	23.5	28.7	32.1	37.1	38.9
	(24.6, 27.8)	(29.8, 33.2)	(35.4, 38.7)	(41.6, 44.5)	(41.8, 45.0)	(19.2, 22.5)	(23.6, 27.8)	(25.2, 29.0)	(30.0, 33.1)	(32.5, 35.9)	(22.3, 24.7)	(27.3, 30.1)	(30.7, 33.6)	(35.8, 38.5)	(37.5, 40.4)
Total energy expenditure (kCal/d)	1715	2341	2572	2453	2185	1851	2706	3327	3110	2713	1782	2517	2945	2790	2443
	(1641, 1789)	(2255, 2424)	(2460, 2673)	(2372, 2532)	(2099, 2274)	(1779, 1923)	(2580, 2821)	(3177, 3461)	(2986, 3222)	(2580, 2845)	(1732, 1834)	(2436, 2594)	(2842, 3045)	(2699, 2876)	(2353, 2534)
Basal metabolic rate (kCal/d)^2^	1049	1400	1462	1431	1330	1099	1596	1894	1803	1588	1075	1495	1677	1622	1455
	(1013, 1084)	(1369, 1431)	(1426, 1498)	(1400, 1462)	(1297, 1361)	(1068, 1130)	(1543, 1646)	(1846, 1944)	(1758, 1851)	(1545, 1629)	(1051, 1099)	(1462, 1529)	(1631, 1720)	(1581, 1662)	(1421, 1490)
Physical activity (kCal/d)^3^	494	707	853	776	638	566	841	1101	994	855	530	771	975	889	743
	(456, 535)	(647, 767)	(776, 927)	(717, 836)	(568, 707)	(521, 614)	(764, 917)	(996, 1204)	(905, 1082)	(757, 953)	(499, 561)	(724, 822)	(910, 1041)	(831, 943)	(681, 805)
Physical activity level^4^	1.63	1.67	1.76	1.72	1.65	1.69	1.70	1.76	1.73	1.71	1.66	1.69	1.76	1.72	1.68
	(1.60, 1.66)	(1.63, 1.72)	(1.70, 1.81)	(1.67, 1.76)	(1.59, 1.70)	(1.64, 1.73)	(1.65, 1.75)	(1.70, 1.82)	(1.67, 1.79)	(1.64, 1.77)	(1.63, 1.69)	(1.65, 1.72)	(1.72, 1.80)	(1.69, 1.76)	(1.63, 1.72)
Total energy intake (kCal/d)	1507	1650	1672	1588	1553	1600	1913	2152	2121	1961	1555	1779	1908	1861	1751
	(1443, 1572)	(1562, 1739)	(1560, 1787)	(1510, 1670)	(1469, 1636)	(1543, 1660)	(1813, 2014)	(2030, 2276)	(2006, 2236)	(1839, 2085)	(1510, 1598)	(1710, 1846)	(1820, 1999)	(1779, 1942)	(1670, 1834)
Carbohydrate intake (% energy)	52.2	50.8	47.5	44.7	45.4	51.3	52.2	47.0	45.4	44.4	51.7	51.5	47.3	45.0	44.9
	(51.2, 53.2)	(49.8, 51.8)	(45.9, 49.2)	(43.1, 46.3)	(43.7, 47.0)	(50.3, 52.2)	(51.1, 53.2)	(45.6, 48.4)	(43.8, 47.0)	(42.6, 46.1)	(51.1, 52.4)	(50.7, 52.2)	(46.2, 48.4)	(43.9, 46.2)	(43.7, 46.1)
Fat intake (% energy)	33.6	34.7	33.4	34.5	34.6	34.1	33.1	32.7	33.0	34.1	33.8	33.9	33.1	33.7	34.4
	(32.6, 34.6)	(33.7, 35.6)	(32.2, 34.7)	(33.1, 35.9)	(33.0, 36.2)	(33.1, 35.0)	(33.1, 34.1)	(31.4, 34.0)	(31.7, 34.3)	(32.6, 35.6)	(33.2, 34.5)	(33.2, 34.6)	(32.2, 34.0)	(32.8, 34.7)	(33.3, 35.4)
Protein intake (% energy)	14.2	14.5	15.8	16.8	17.7	14.6	14.7	16.0	16.0	16.9	14.4	14.6	15.9	16.4	17.3
	(13.7, 14.7)	(13.9, 15.1)	(14.9, 16.7)	(16.0, 17.6)	(16.9, 18.4)	(14.2, 15.1)	(14.1, 15.3)	(15.1, 16.8)	(15.3, 16.8)	(16.1, 17.7)	(14.1, 14.8)	(14.2, 15.0)	(15.3, 16.5)	(15.8, 16.9)	(16.8, 17.9)
Alcohol intake (% energy)	0.0	0.0	3.2	4.0	2.4	0.0	0.0	4.3	5.6	4.6	0.0	0.00	3.7	4.8	3.5
	(0.0, 0.0)	(0.0, 0.0)	(1.7, 4.6)	(2.8, 5.3)	(1.4, 3.4)	(0.0, 0.0)	(0.0, 0.0)	(2.7, 5.9)	(4.0, 7.1)	(2.9, 6.2)	(0.0, 0.0)	(0.0, 0.1)	(2.7, 4.8)	(3.8, 5.8)	(2.5, 4.4)

1 Data are *n* or mean (95% confidence interval).

2 Basal metabolic rate calculated using Schofield equations.

3 Physical activity energy expenditure calculated as (0.9 total energy expenditure) − basal metabolic rate.

4 Physical activity level is calculated as total energy expenditure/basal metabolic rate.

**TABLE 2:** Energy intake, total energy expenditure, and measurement error in the UK National Diet and Nutrition Survey doubly labeled water participants (2008–2015)^1^.

Age group (y)	Sex	Years 2008–2011					Years 2013–2015					Combined years				
		*n*	EI (kcal/d)	TEE (kcal/d)	TEE-EI (kcal/d)	EI:TEE	*n*	EI (kcal/d)	TEE (kcal/d)	TEE-EI (kcal/d)	EI:TEE	*n*	EI (kcal/d)	TEE (kcal/d)	TEE-EI (kcal/d)	EI:TEE
4–10	Male	41	1610	1843	233	0.9	33	1566	1862	297	0.86	74	1590	1852	262	0.88
			(1524, 1696)	(1729, 1957)	(117, 348)	(0.84, 0.96)		(1488, 1642)	(1782, 1943)	(186, 409)	(0.8, 0.91)		(1533, 1648)	(1780, 1923)	(182, 341)	(0.84, 0.92)
	Female	41	1552	1763	211	0.91	32	1426	1655	228	0.89	73	1497	1715	218	0.9
			(1466, 1638)	(1663, 1863)	(89, 332)	(0.84, 0.98)		(1326, 1526)	(1541, 1769)	(108, 349)	(0.81, 0.96)		(1432, 1562)	(1641, 1790)	(134, 302)	(0.85, 0.95)
	Total	82	1581	1803	222	0.91	65	1497	1760	263	0.87	147	1544	1784	240	0.89
			(1521, 1641)	(1728, 1878)	(140, 304)	(0.86, 0.95)		(1433, 1560)	(1688, 1833)	(183, 344)	(0.83, 0.92)		(1500, 1587)	(1732, 1836)	(183, 298)	(0.86, 0.92)
11–15	Male	34	2058	2714	656	0.78	42	1775	2705	930	0.68	76	1901	2709	807	0.73
			(1928, 2188)	(2526, 2902)	(475, 837)	(0.72, 0.84)		(1637, 1912)	(2557, 2852)	(729, 1130)	(0.62, 0.74)		(1803, 2000)	(2594, 2823)	(670, 945)	(0.68, 0.77)
	Female	38	1712	2379	666	0.74	42	1575	2307	732	0.7	80	1640	2341	701	0.72
			(1596, 1828)	(2252, 2506)	(492, 840)	(0.68, 0.81)		(1444, 1705)	(2192, 2422)	(574, 890)	(0.64, 0.76)		(1553, 1728)	(2257, 2425)	(586, 815)	(0.68, 0.77)
	Total	72	1875	2537	661	0.76	84	1675	2506	831	0.69	156	1767	2520	753	0.72
			(1781, 1969)	(2421, 2652)	(539, 784)	(0.72, 0.81)		(1580, 1770)	(2404, 2607)	(704, 958)	(0.65, 0.73)		(1699, 1836)	(2445, 2596)	(664, 841)	(0.69, 0.75)
16–49	Male	38	2262	3462	1201	0.67	51	2052	3231	1179	0.65	89	2142	3330	1188	0.66
			(2070, 2454)	(3240, 3685)	(920, 1461)	(0.61, 0.73)		(1892, 2213)	(3066, 3397)	(981, 1377)	(0.6, 0.71)		(2019, 2264)	(3197, 3464)	(1032, 1344)	(0.62, 0.7)
	Female	40	1609	2530	921	0.65	51	1709	2606	898	0.68	91	1665	2573	908	0.67
			(1469, 1749)	(2385, 2675)	(762, 1079)	(0.59, 0.7)		(1535, 1883)	(2464, 2749)	(663, 1133)	(0.61, 0.76)		(1551, 1779)	(2472, 2673)	(761, 1054)	(0.62, 0.72)
	Total	78	1927	2984	1057	0.66	102	1881	2919	1038	0.67	180	1901	2947	1046	0.66
			(1790, 2064)	(2818, 3151)	(907, 1208)	(0.62, 0.7)		(1760, 2002)	(2795, 3043)	(885, 1192)	(0.62, 0.72)		(1811, 1991)	(2848, 3047)	(939, 1154)	(0.63, 0.7)
50–64	Male	41	2160	3148	999	0.7	42	2065	3074	1009	0.69	83	2112	3111	999	0.69
			(2020, 2300)	(2972, 3324)	(805, 1171)	(0.65, 0.74)		(1879, 2251)	(2920, 3228)	(780, 1237)	(0.63, 0.75)		(1997, 2227)	(2996, 3225)	(855, 1142)	(0.66, 0.73)
	Female	37	1588	2432	844	0.67	42	1577	2474	897	0.65	79	1582	2454	872	0.66
			(1486, 1690)	(2312, 2551)	(701, 987)	(0.62, 0.72)		(1454, 1701)	(2362, 2586)	(752, 1041)	(0.6, 0.7)		(1503, 1662)	(2374, 2534)	(772, 972)	(0.62, 0.69)
	Total	78	1888	2808	920	0.68	84	1821	2774	953	0.67	162	1854	2790	937	0.68
			(1780, 1996)	(2675, 2942)	(804, 1036)	(0.65, 0.72)		(1700, 1943)	(2660, 2888)	(820, 1086)	(0.63, 0.71)		(1773, 1935)	(2704, 2877)	(849, 1025)	(0.65, 0.7)
65+	Male	29	1900	2661	762	0.73	32	2000	2763	763	0.73	61	1952	2715	762	0.73
			(1737, 2062)	(2479, 2844)	(590, 934)	(0.67, 0.78)		(1812, 2189)	(2568, 2959)	(878, 939)	(0.68, 0.79)		(1830, 1075)	(2584, 28460	(643, 882)	(0.69, 0.77)
	Female	32	1548	2163	615	0.73	32	1541	2212	671	0.72	64	1544	2187	643	0.72
			(1431, 1665)	(2035, 2291)	(467, 763)	(0.67, 0.79)		(1413, 1668)	(2088, 2336)	(504, 839)	(0.64, 0.8)		(1460, 1628)	(2101, 2274)	(534, 752)	(0.68, 0.77)
	Total	61	1715	2400	685	0.72	64	1770	2488	717	0.73	125	1743	2445	701	0.73
			(1609, 1821)	(2276, 2524)	(574, 796)	(0.69, 0.77)		(1646, 1895)	(2355, 2620)	(599, 836)	(0.68, 0.77)		(1662, 1825)	(2355, 2536)	(621, 782)	(0.7, 0.76)

Abbreviations: EI, energy intake; TEE, total energy expenditure.

1 Data are *n* or mean (95% confidence interval).

There were few significant differences between survey years; all P values for the different age/sex strata are given Supplemental Table 1. Only males aged 16 to 49 y had both a lower EI (*P* = 0.02) in 2013–2015 (mean: 2064 kcal/d; 95% CI: 1901, 2224 kcal/d) compared with 2271 kcal/d (95% CI: 2080, 2465 kcal/d) in 2008–2011 and a lower TEE (mean: 3229 kcal/d; 95% CI: 3064, 3394 kcal/d; *P* = 0.02) in 2013–2014 compared to 2008–2011 (mean: 3461 kcal/d; 95% CI: 3239, 3683 kcal/d). For boys aged 11 to 15 y, we saw a lower reported EI (mean: 2262 kcal/d; 95% CI: 2070, 2454 kcal/d compared with 1775 kcal/d; 95% CI: 1637, 1912 kcal/d, 2008–2011 and 2013–2015 respectively), whereas the TEE was marginally higher (2714 kcal/d; 95% CI: 2526, 2902 kcal/d) in 2008–2011 compared with 2705 kcal/d (95% CI: 2557, 2852 kcal/d) in 2013–2015. For all other groups, there were no generalizable or significant changes in either EI or TEE across the 2 study periods.

Likewise, for the majority of the age/sex strata, the EI:TEE ratio was similar across the measurement years (Table 2), except for boys aged 11 to 15 y, in whom the EI:TEE ratio was lower in 2013–2014. The higher observed underreporting in boys aged 11 to 15 y (22%; 95% CI: 28, 16% compared with 32%; 95% CI: 38, 26%, survey years 2008–2011 and survey years 2013–2015, respectively) appears to have been due to a lower estimated EI from food diary records rather than any difference in measured TEE (Table 2). In Table 3, we show that the EI:TEE ratio for survey years 2008–2011 and survey years 2013–2015 was generally <1 in all age strata, indicating a significant level of underestimation of EI based on the dietary assessment. The EI:TEE ratio for separate age strata showed the best agreement in children, at 0.89 (95% CI: 0.86, 0.92) (Table 2). Although EI:TEE ratio indicated a significant level of underestimation, overestimation of energy intake was also evident, particularly in the 4 to 10 y age stratum (Supplemental Figure 2).

**TABLE 3:** Associations with measurement error in the UK National Diet and Nutrition Survey doubly labeled water participants (2008–2015).

	Sample size (*n*)	EI:TEE coefficient (95% CI)	TEE-EI coefficient (kcal) (95% CI)
Substudy years			
2008–2011	371	Reference	Reference
2013–2015	399	−0.04 (−0.07, 0.00)	78.1 (−3.6, 160)
Age (per year)	770	0.001 (−0.0007, 0.0025)	−8.4 (−1.2, −4.8)
Sex			
Male	383	Reference	Reference
Female	387	0.007 (−0.021, 0.035)	−173.4 (−252.7, −93.9)
Government Office Region			
North East	38	Reference	Reference
North West	78	−0.02 (−0.09, 0.05)	19.1 (−190.4, 228.3)
Yorkshire and the Humber	63	−0.02 (−0.10, 0.06)	80 (−143.3, 303.1)
East Midlands	80	−0.001 (−0.08, 0.07)	27.2 (−184.2, 238.6)
West Midlands	62	−0.03 (−0.11, 0.05)	49.7 (−173.4, 272.8)
East of England	63	0.02 (−0.06, 0.10)	−39.6 (−258.7, 179.4)
London	53	0.05 (−0.03, 0.14)	−85 (−329.9, 159.1)
South East	70	−0.05 (−0.13, 0.02)	140.7 (−73.1, 354.7)
South West	61	−0.05 (−0.13, 0.03)	136.6 (−85, 358.3)
Wales	72	0.003 (−0.07, 0.08)	31.8 (−185.3, 248.6)
Scotland	50	−0.002 (−0.08, 0.08)	18.4 (−214.2, 250.8)
Northern Ireland	80	−0.02 (−0.09, 0.06)	99.4 (−113.7, 312.2)
Ethnic group			
White	717	Reference	Reference
Mixed	17	−0.01 (−0.10, 0.09)	70.5 (−192.5, 333.4)
Black	8	−0.04 (−0.18, 0.10)	54.2 (−332.2, 440.7)
Asian	24	0.07 (−0.01, 0.15)	−191.6 (−423.5, 40.4)
Other	4	−0.01 (−0.19, 0.17)	−162.9 (−681.2, 355.6)
Income (per £1000)	697	−0.0003 (−0.0011, 0.0004)	0.5 (−1.7, 2.6)
BMI (per kg/m^2^)	770	−0.017^1^ (−0.020, −0.014)	55.7 (47.1, 64.3)
Education (≥16 y)			
Degree or equivalent	94	Reference	Reference
Higher education, below degree	36	0.01 (−0.06, 0.08)	−37 (−243.6, 169.6)
A-levels or equivalent	60	−0.01 (−0.07, 0.05)	45.6 (−133.3, 224)
GCSE	105	−0.003 (−0.06, 0.05)	−31.3 (−167, 152.1)
Foreign or other	18	0.05 (−0.05, 0.15)	−88.1 (−376.2, 200.2)
No qualifications	99	−0.003 (−0.07, 0.06)	14.3 (−166.2, 194.7)
Still in full-time education	352	0.01 (−0.06, 0.08)	−171.5 (−373.8, 30.8)
Smoking status			
Non-smoker	656	Reference	Reference
Smoker	107	−0.01 (−0.06, 0.03)	20.8 (−106.8, 148.1)
Drinking of alcohol frequency (≥8 y)			
≥5 d/wk	65	Reference	Reference
3 or 4 d/wk	60	−0.07 (−0.14, 0.00)	202.5 (7.9, 397.2)
Once or twice a week	123	−0.06 (−0.12, 0.00)	183 (10.3, 355.6)
Once or twice a month	88	−0.09^1^ (−0.16, −0.02)	229.3 (37.5, 421.3)
Once every couple of months	60	−0.09^1^ (−0.17, −0.02)	187.3 (−16.7, 391)
Once or twice a year	81	−0.13^1^ (−0.20, −0.05)	240.5 (37.7, 443.3)
Never in the last 12 mo	285	−0.06 (−0.12, 0.01)	55.7 (−133.3, 244.6)

Abbreviations: CI, confidence interval; EI, energy intake; GCSE, general certificate of secondary education; TEE, total energy expenditure.

1 Significant association (*P* < 0.05) using multiple regression.

To assess the representativeness of the DLW sample used in the present analysis, in relation to the overall survey sample (for respective years when the DLW substudies were conducted), plots were created for 5 variables of interest: BMI (kilograms per meters squared) (Supplemental Figure 3), EI (kcal per day) (Supplemental Figure 4), total fruit and vegetables consumption (grams per day) (Supplemental Figure 5), free sugars intake (percentage of total energy) (Supplemental Figure 6), and saturated fatty acid intake (percentage of total energy) (Supplemental Figure 7). All dietary variables were determined from the unweighed 4-d food diary. Supplementary Figures 3–7 do not show any clear differences between the DLW subsamples and the overall survey sample responses, indicating that the DLW subsamples were representative of the overall NDNS sample with respect to these measures.

**FIGURE 1:**
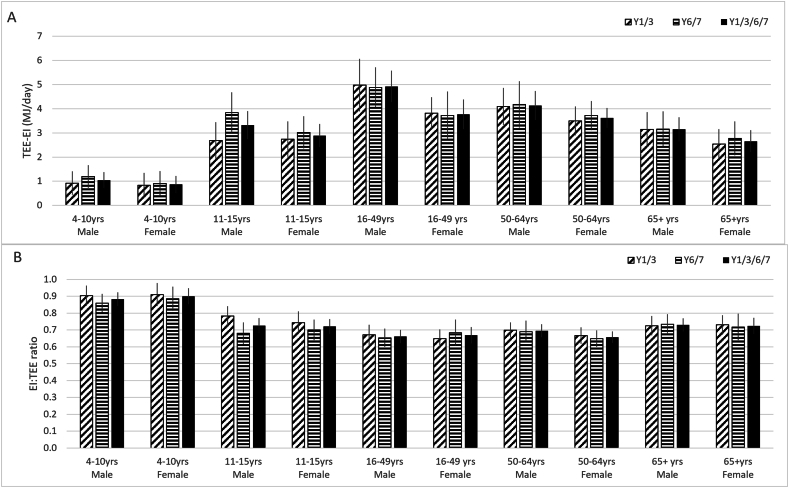
Mean difference [total energy expenditure (TEE)–energy intake (EI)] (MJ/d) (A) and mean ratio (EI:TEE) (B) by age and sex with 95% confidence intervals for the National Diet and Nutrition Survey years 2008–2011 (diagonal stripes), years 2013–2015 (horizontal stripes), and combined years 2008–2015 (solid black). Y, year.

The mean differences between the EI and TEE did not change between survey years in any of the age/sex strata, and so further analysis of the DLW subsample data was carried out on the dataset with sexes combined. Separate survey year groups and sex-combined datasets are shown in Table 2. The combined datasets indicate that the mean difference between TEE and EI was lowest in the 4 to 10 y age group at 229 kcal/d (95% CI: 172, 287 kcal/d), highest in the 16 to 49 y age group (1037 kcal/d; 95% CI: 929, 1144 kcal/d), and lower in the older age groups (Table 3 and Figure 1A). The association between the mean difference and age was also confirmed with multiple linear regression analyses (Table 3), which showed a decrease of −8.4 kcal/y (95% CI: −1.2, −4.8 kcal/y). The EI:TEE ratios of the strata followed a similar pattern, being highest for ages 4 to 10 y (0.89; 95% CI: 0.86, 0.92) and lowest for ages 16 to 49 y (0.66; 95% CI: 0.63, 0.70), equating to an underreporting of 11% and 34%, respectively, for EI (Table 3 and Figure 1B). However, the EI:TEE ratio was not associated with age in the multiple linear regression analyses at 0.001 per year (95% CI: −0.0007, 0.0025) (Table 3).

**FIGURE 2:**
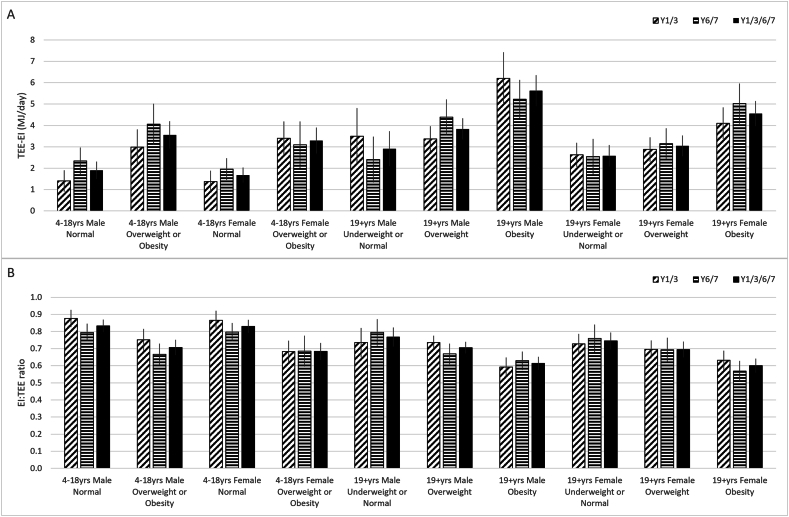
Mean difference [total energy expenditure (TEE)–energy intake (EI)] (MJ/d) (A) and mean ratio (EI:TEE) (B) by age/sex and BMI with 95% confidence intervals for the National Diet and Nutrition Survey years 2008–2011 (diagonal stripes), years 2013–2015 (horizontal stripes), and combined years 2008–2015 (solid black). Y, year.

There was a significant inverse association between both EI:TEE ratio and mean difference and BMI, such that the level of underestimation of EI increased with increasing BMI (Figure 2A, B), independent of age. These findings were confirmed with multiple linear regression analyses (Table 3), which showed a negative association of −0.02 EI:TEE ratio units/kg/m^2^ (95% CI: −0.020, −0.014 EI:TEE ratio units/kg/m^2^) and a positive association of the mean difference of 55.7 kcal/kg/m^2^ (95% CI: 47.1, 64.3 kcal/kg/m^2^).

Female sex was associated with a −173.4 kcal (95% CI: −252.7, −93.9 kcal) greater mean difference between TEE and EI than the males, but no difference in EI:TEE ratio. We found no association in mean difference or EI:TEE ratio with ethnicity, education, equivalized income or geography.

## Discussion

There is evidence of measurement error within the UK NDNS, characterized here by an overall underestimation of EI of approximately 27% (95% CI: 25, 28%), determined from the self-reported dietary data. We have observed that, although underestimation of EI is significant in all age/sex strata, there does appear to be a lesser degree of measurement error in estimated EI in young children than in adults.

Our findings are comparable to wider research findings, not just from national surveys, that higher BMI [[25], [26], [27], [28], [29], [30], [31], [32], [33]] and age [[27], [28], [29], [30]] are commonly associated with a higher degree of measurement error in the estimation of EIs through dietary assessment, namely underestimation.

In most surveys, the evaluation of measurement error is made using one of two methods based on the 95% confidence limits: either agreement between the ratio of EI:BMR along with a respective PAL, usually 1.55 (Goldberg cutoff) [6,7], or of the expected ratio of EI:estimated energy requirements proposed by Huang et al. [31]. These two methods allow for an individual to be defined as an underreporter or overreporter and not, as in our study, to allow for the degree of measurement error to be objectively determined. Using these methods, data from the United States NHANES 2003–2012 showed a higher prevalence of underreporting (25.7%) in adults [27] compared with children and adolescents [28] (13.1%). In Europe, underreporting of EI has been shown as comparable to that in the United States, with 33% of adolescents underreporting [32] and 9.5% of children and infants underreporting [33]. Although these methods do not allow for the degree of measurement error to be determined, they do indicate that measurement error of total EI derived using self-reported measures of dietary intake is highly prevalent.

The Observing Protein and Energy Nutrition study in the United States [34] found that, in comparison with TEE measured by DLW, men aged 40 to 69 y underreported EI by 12% to 14%, whereas females underreported EI by 16% to 20% when using 2 24-h recalls. Similar sex differences were also observed when using the food frequency questionnaire method. A recent meta-analysis by McKenzie et al. [34], which included 11 small DLW studies using estimated food records, reported that males significantly underestimate self-reported EI to a greater extent than females by 41 kcal/d. These results are consistent with our data that show males underestimate EI on average by −173 kcal (95% CI: −252, −94 kcal) more than the females.

We determined whether there had been any differences in measurement error between the two periods and found that the measurement error for the combined datasets did not differ significantly between the EI:TEE ratios of the two survey year periods (0.75; 95% CI: 0.73, 0.77 compared with 0.72; 95% CI: 0.69, 0.74, 2008–2011 and 2013–2015, respectively).

Our analysis indicated that the average TEE decreased by 231 kcal/d (6.7%) (*P* = 0.02) in males aged 16 to 49 y between the survey years. This is in agreement with the recent study of Speakman et al. [35] in which they showed that when adjusted for body size and composition, TEE in males decreased by 222 kcal/d (7.7%) across a time period of approximately three decades. It is important to note that males in the Speakman et al. [1] study were included in the analysis if they were aged ≥18 y and not restricted to a specific age stratum as in our analysis. They also found a significant decrease of 122 kcal/d (5.6%) in adjusted TEE over time in the females. Although we did not observe a significant difference for females aged 16 to 49 y (*P* = 0.43, difference 76 kcal/d), it may be that our data set is underpowered to detect such a change, as Speakman et al. [1] used data from the IAEA DLW database consisting of 4799 adults, which includes data collected over a longer time period (3 decades).

Our work has several strengths; the NDNS is designed to be nationally representative with no observed selection bias for the DLW subsample. The survey included DLW, which is the reference method for TEE measurement, and repeated substudies conducted within each 5-y survey round. Furthermore, the NDNS used a robust detailed quantitative 4-d diet diary to assess EI.

There are also a number of limitations with this study. The TEE measurement is based on 10 d, whereas the diet diary only covers 4 d. We have observed that in the NDNS DLW substudies, EI differs by day of the week, being higher during the weekend compared with weekdays (data not shown); this difference in EI between days can be as high as 239 kcal/d. Because the measurement of TEE always covered ≥1 weekend day and the assessment of EI may not have been done, the question remains as to whether the difference in timing of measurements could explain some of the differences between reported EI and TEE.

There are a variety of factors contributing to measurement error, both in the practice of self-reported dietary assessment to provide estimates of intake and in the assessment and measurement of such error through the DLW protocol. In dietary assessment, factors include conscious and unconscious bias in reporting, including modified behavior and undereating [36,37] and in the reporting of foods and amounts consumed. Cognition, memory performance, and literacy can also affect recall and reporting; factors apply both at the meal (e.g., forgotten snacks) and individual item level, portion size estimation, and food composition, all of which can be subject to bias. Furthermore, such errors can arise from participant engagement, motivation, and burden. In the same way, we cannot be certain that participants maintained their usual behavior during the DLW protocol. In the NDNS, attempts were made to mitigate underreporting by minimizing the impact of some of these factors through training of interviewers, instructions to survey participants, and careful design of survey materials. Error can also arise from research methods, spanning data collection (design, structure, and application of dietary assessment tools and protocols) and approaches for dietary data processing, coding, and food composition. Portion sizes and food composition data of foods and dishes were updated whenever new information became available. Dietary coding and portion estimation is informed by the level of detail provided by participants in the food diaries and necessarily pragmatic. Food composition databases contain average or estimated values, including those for energy concentration, and there are likely to be variations that have not been included in the estimation of EI. It might be possible that these efforts to reduce measurement error are effective but not sufficient to significantly decrease the error. The quantification of measurement error, as presented in this study for EI, is crucial in helping to understand the limitations of research and the further development of improved methods. With the limited number of DLW studies available in the literature, our work will contribute evidence that will help other and future surveys. However, it is important to keep in mind that such assessment is also limited; in respect of error in EI, for example, it does not follow that this tells us about the error or its magnitude for nutrient intakes more broadly, or in relation to intakes for specific foods or food groups.

In conclusion, the 2 DLW substudies in the UK NDNS have allowed quantification of the existence of measurement error in estimated total EI from the dietary assessment. We demonstrated that this has remained predominantly similar over the two measurement periods carried out between 2008 and 2015 at approximately 27%, with the variability dependent on sex, age (children compared with adults), and BMI. Further work is needed to understand the factors associated with measurement error, both in respect to cause and impact. Although the DLW studies have determined the level at which measurement error in estimated EI exists within the NDNS, there is as yet no obvious approach to define which parts of the diet are ‘misreported,’ the impact of this measurement error on other nutrients and specific foods, or where other errors in the calculation of EI occur. Therefore, nutrient intakes are not adjusted in NDNS reported results. The field of dietary assessment continues to evolve, and the current pace of development in AI technologies may soon offer potential to considerably improve research methods and data quality, both in respect of completeness, detail, and accuracy of assessment. That said, substantive work is still needed to incorporate such advances and then to develop, validate, and demonstrate efficacy and feasibility for population research. In the meantime, dietary assessment in the free-living, carried out at the population level and at scale, remains reliant on pragmatic methods and self-reported estimates of foods and drinks consumed. Our research shows that NDNS provides robust and valuable nutrient and diet data with an objective assessment of its measurement error.

## Author contributions

The authors’ responsibilities were as follows – MCV, CR, PP: designed research; MCV, DG, ERO, DC: conducted research; DC: analyzed data; MCV, CR, AK, PP: wrote the article; AK, NJW, PP: had primary responsibility for final content; and all authors: read and approved the final manuscript.

## Data availability

Data described in the article are publicly and freely available without restriction at the UK data service (https://ukdataservice.ac.uk/); code book and analytic code will be made available upon request to the corresponding author. For the purpose of open access, the author has applied a Creative Commons Attribution (CC BY) license to any author-accepted manuscript version arising.

## Funding

Original data collection for the core National Diet and Nutrition Survey (NDNS) was jointly funded by Public Health England (PHE) and the UK Food Standards Agency. Devolved government departments in Scotland, Wales, and Northern Ireland funded additional NDNS recruitment. The NDNS (2008–2019) was carried out by a consortium comprising the National Centre for Social Research and the Medical Research Council Elsie Widdowson Laboratory with fieldwork in Northern Ireland carried out by the Northern Ireland Statistics and Research Agency. This secondary analysis was led by the Nutrition Measurement Platform at the Medical Research Council (MRC) Epidemiology Unit, supported by the National Institute for Health and Care Research (NIHR) Cambridge Biomedical Research Centre (NIHR203312). The views expressed are those of the authors and not necessarily those of the NIHR, MRC, or PHE.

## Conflict of interest

The authors report no conflicts of interest.
