# Supplementary material for: Measurement error in total energy intake in the United Kingdom National Diet and Nutrition Survey (2008–2015)
Source: Am J Clin Nutr. 2025 Sep 24;122(6):1819–28. doi: 10.1016/j.ajcnut.2025.09.037 (PMC12799365; doi:10.1016/j.ajcnut.2025.09.037)
Supplement: multimedia component 1 [file mmc1.docx]

**Measurement error in total energy intake in the United Kingdom National Diet and Nutrition Survey (2008-2015)**

Michelle C Venables^1^, Caireen Roberts^1^, Dan Griffiths^1^, Elise R Orford^1^, Dave Collins^1^, Albert Koulman^1^, Nicholas J Wareham^1^ and Polly Page^1^

**Author Affiliations:** ^1^Medical Research Council Epidemiology Unit, University of Cambridge, Cambridge, UK

**Authors’ last names:** Venables, Roberts, Griffiths, Orford, Collins, Koulman, Wareham, Page

**Corresponding Author:** Albert Koulman, MRC Epidemiology Unit, Level 3 Institute of Metabolic Science, University of Cambridge School of Clinical Medicine, Cambridge CB2 0SL; +44(0) 1223 763202; ak675@cam.ac.uk


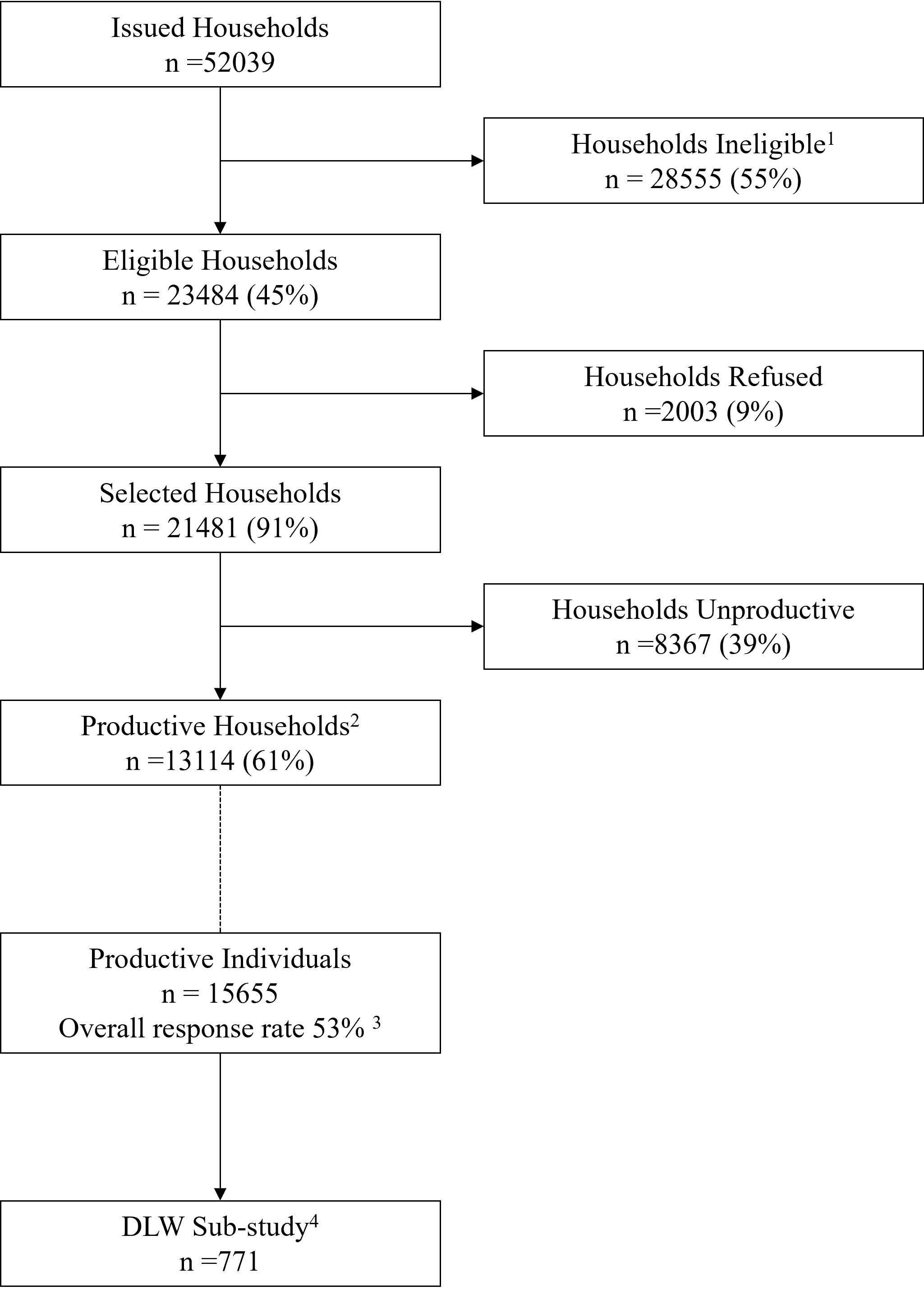


**Supplemental Figure 1:** Flow diagram of recruitment numbers and response rates for the UK National Diet and Nutrition Survey Rolling Programme Years 1–11, 2008–2019. DLW, doubly labelled water. ^1^: The majority were addresses selected for the child boost sample that were screened out because they did not contain any children in the eligible age range (1.5 to 18 years). The remainder included vacant or derelict properties and institutions. ^2^: Those in which one or more participant(s) completed 3 or 4 diary days. ^3^: Response is calculation of: the proportion of eligible addresses that are productive multiplied by the proportion of total individuals that are productive. Productive individuals are those that completed 3 or 4 diary days. ^4^: Included in Years 1, 3, 6 and 7 for participants aged 4 years and above only. Futher details in Venables MC, Roberts C, Nicholson S, Bates B, Jones KS, Ashford R, Hill S, Farooq A, Koulman A, Wareham NJ, et al. Data Resource Profile: United Kingdom National Diet and Nutrition Survey Rolling Programme (2008-19). Int J Epidemiol 2022. doi: 10.1093/ije/dyac106.

**Supplemental Table 1.** P-values for the difference between study periods for Energy Intake (EI), Total Energy Expenditure (TEE) and Measurement error (EI:TEE) in the UK National Diet and Nutrition Survey Rolling Programme doubly labelled water participants (2008-2015)

| Age group |  | EI | TEE | EI:TEE |
| --- | --- | --- | --- | --- |
| 4-10yrs | Male | 0.66 | 0.86 | 0.32 |
|  | Female | 0.22 | 0.31 | 0.59 |
| 11-15yrs | Male | 0.01 | 0.93 | 0.02 |
|  | Female | 0.16 | 0.48 | 0.32 |
| 16-49yrs | Male | 0.03 | 0.02 | 0.66 |
|  | Female | 0.28 | 0.43 | 0.37 |
| 50-64yrs | Male | 0.32 | 0.46 | 0.83 |
|  | Female | 0.91 | 0.68 | 0.66 |
| 65+yrs | Male | 0.37 | 0.38 | 0.84 |
|  | Female | 0.95 | 0.67 | 0.79 |


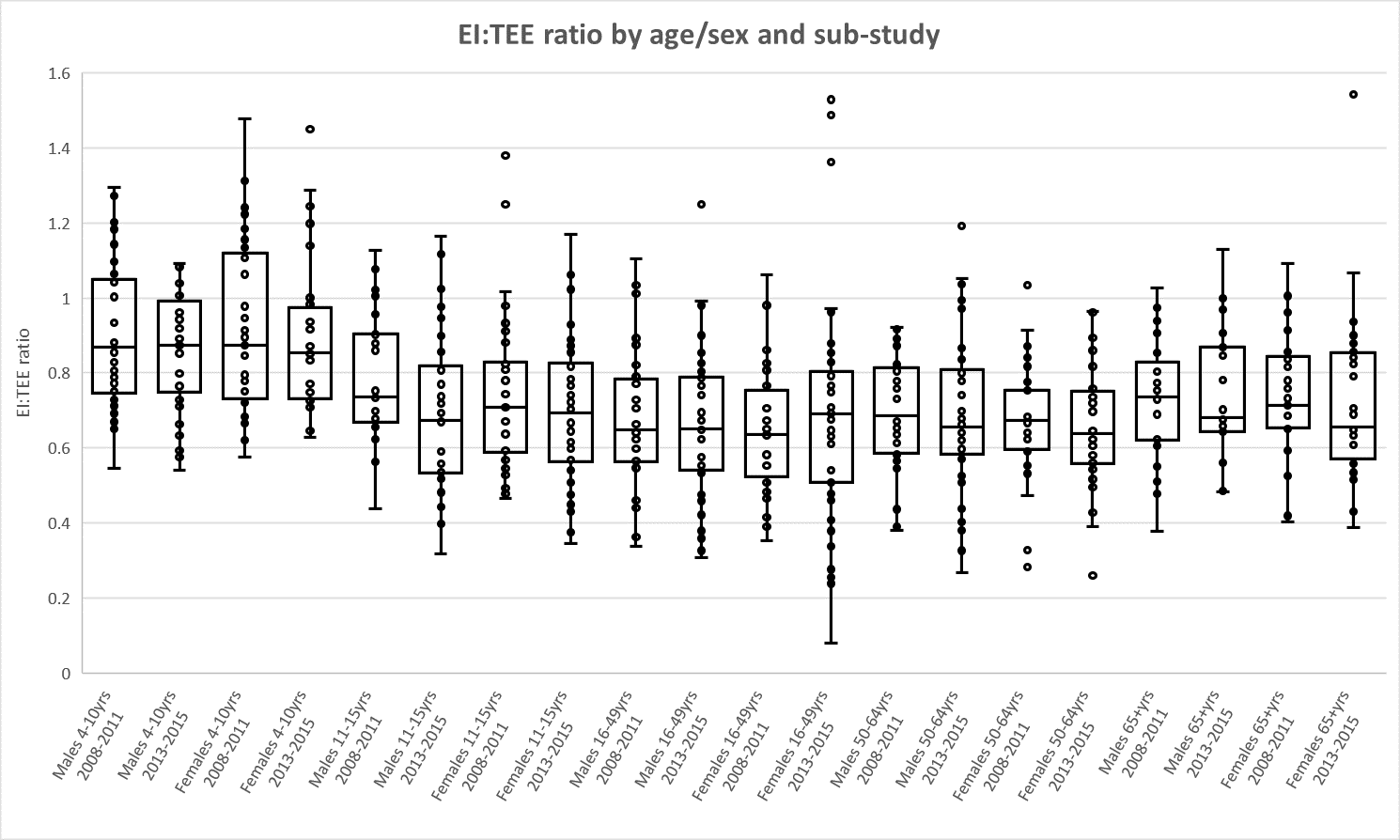


**Supplemental Figure 2:** Misreporting (EI:TEE ratio) across all age and sex strata, for all doubly labelled water participants within the UK National Diet and Nutrition Survey Rolling Programme, during the measurement periods 2008-2011 and 2013-2015.

EI, energy intake; TEE, total energy expenditure.


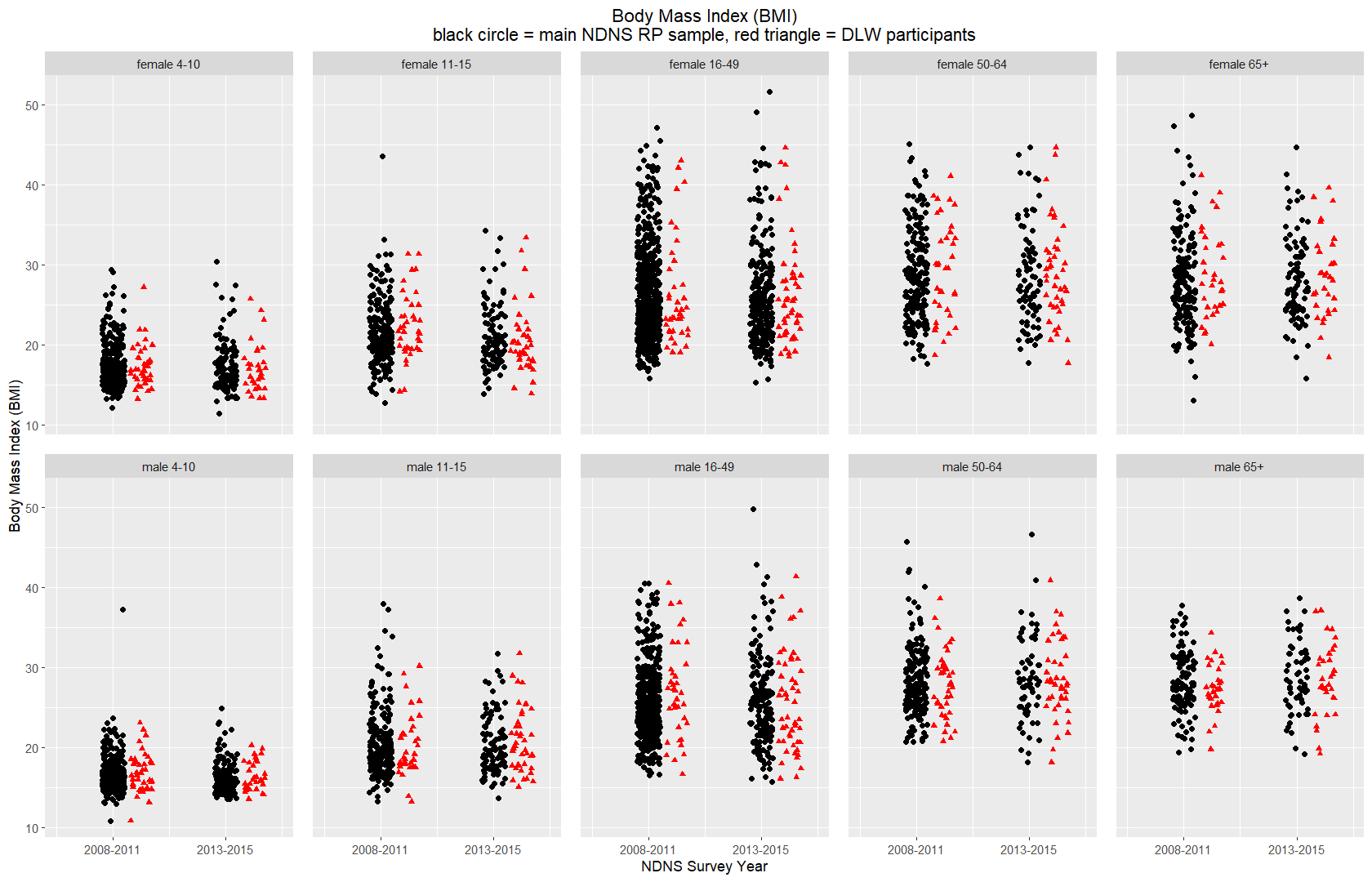


**Supplemental Figure 3:** The comparison of Body Mass Index (BMI) across all age/sex strata, for doubly labelled water participants *vs.* all participants within the main UK National Diet and Nutrition Survey Rolling Programme, during the measurement periods 2008-2011 and 2013-2015.

Black circles denote the main NDNS RP participants; red triangles denote the doubly labelled water participants.


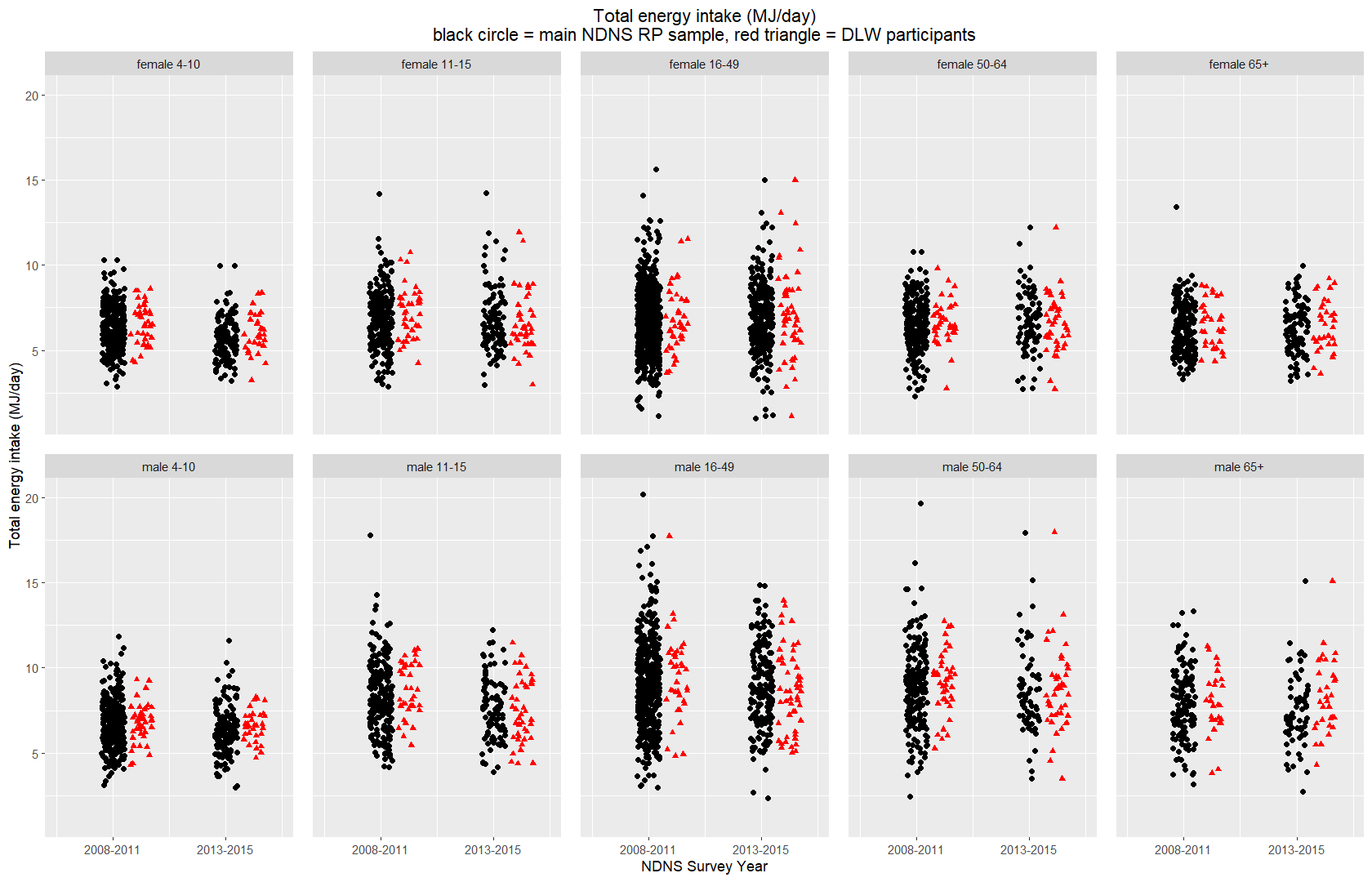


**Supplemental Figure 4:** The comparison of total energy intake across all age/sex strata, for doubly labelled water participants *vs*. all participants within the main UK National Diet and Nutrition Survey Rolling Programme, during the measurement periods 2008-2011 and 2013-2015.

Black circles denote the main NDNS RP participants; red triangles denote the doubly labelled water participants.


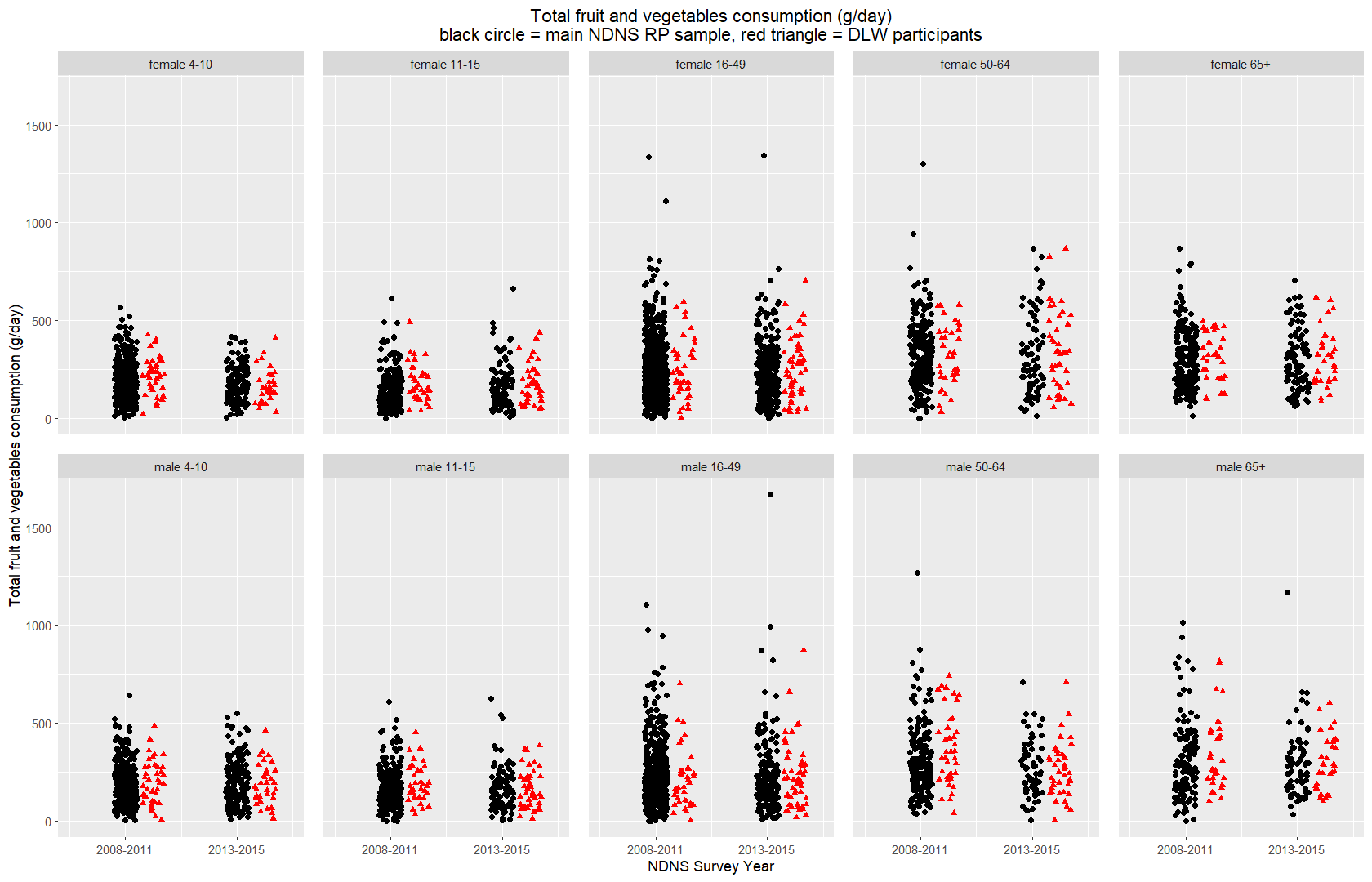


**Supplemental Figure 5:** The comparison of total fruit and vegetable consumption across all age/sex strata, for doubly labelled water participants *vs.* all participants within the main UK National Diet and Nutrition Survey Rolling Programme, during the measurement periods 2008-2011 and 2013-2015.

Black circles denote the main NDNS RP participants; red triangles denote the doubly labelled water participants.


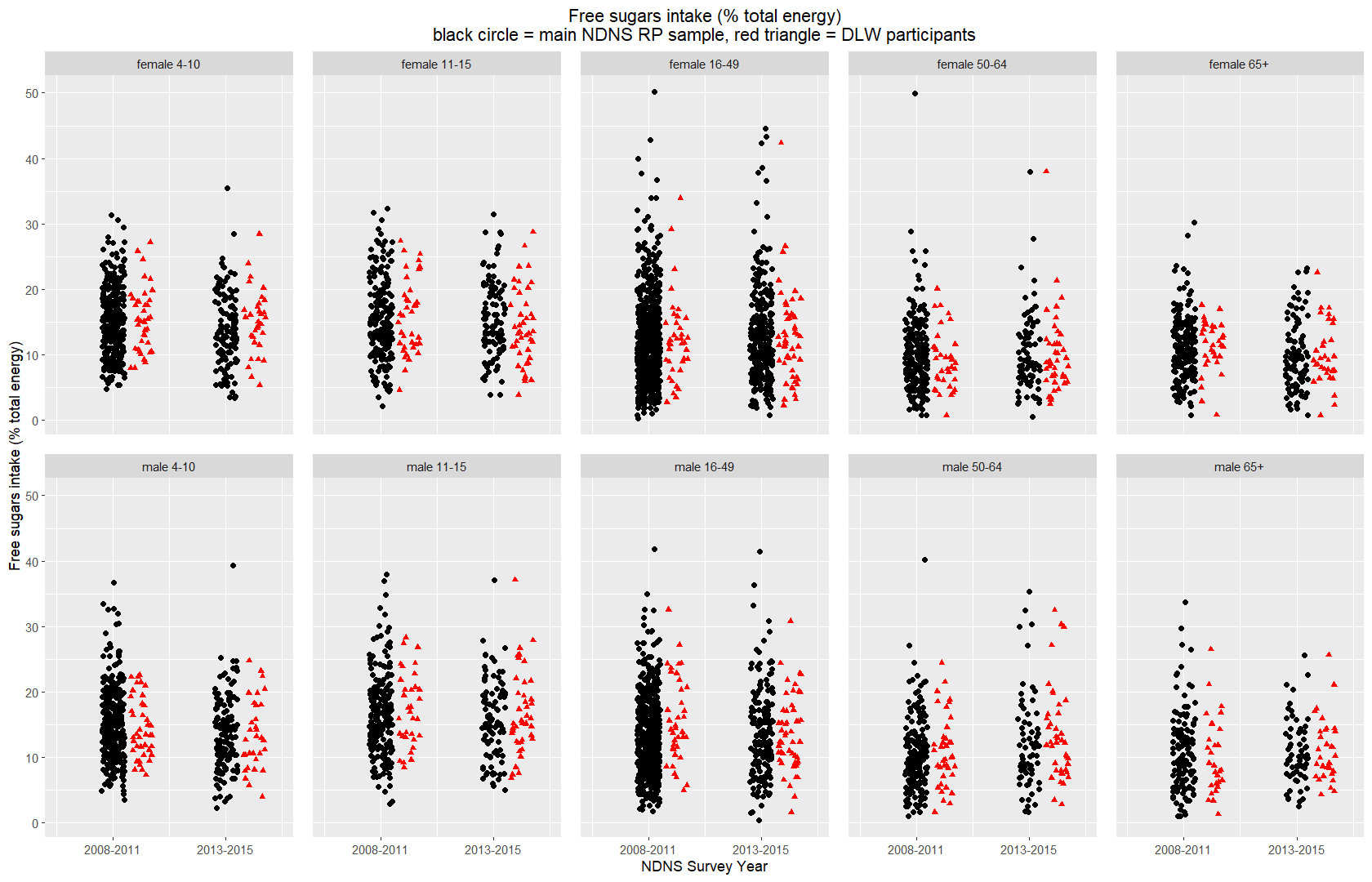


**Supplemental Figure 6:** The comparison of free sugar intake across all age/sex strata, for doubly labelled water participants *vs.* all participants within the main UK National Diet and Nutrition Survey Rolling Programme, during the measurement periods 2008-2011 and 2013-2015.

Black circles denote the main NDNS RP participants; red triangles denote the doubly labelled water participants.


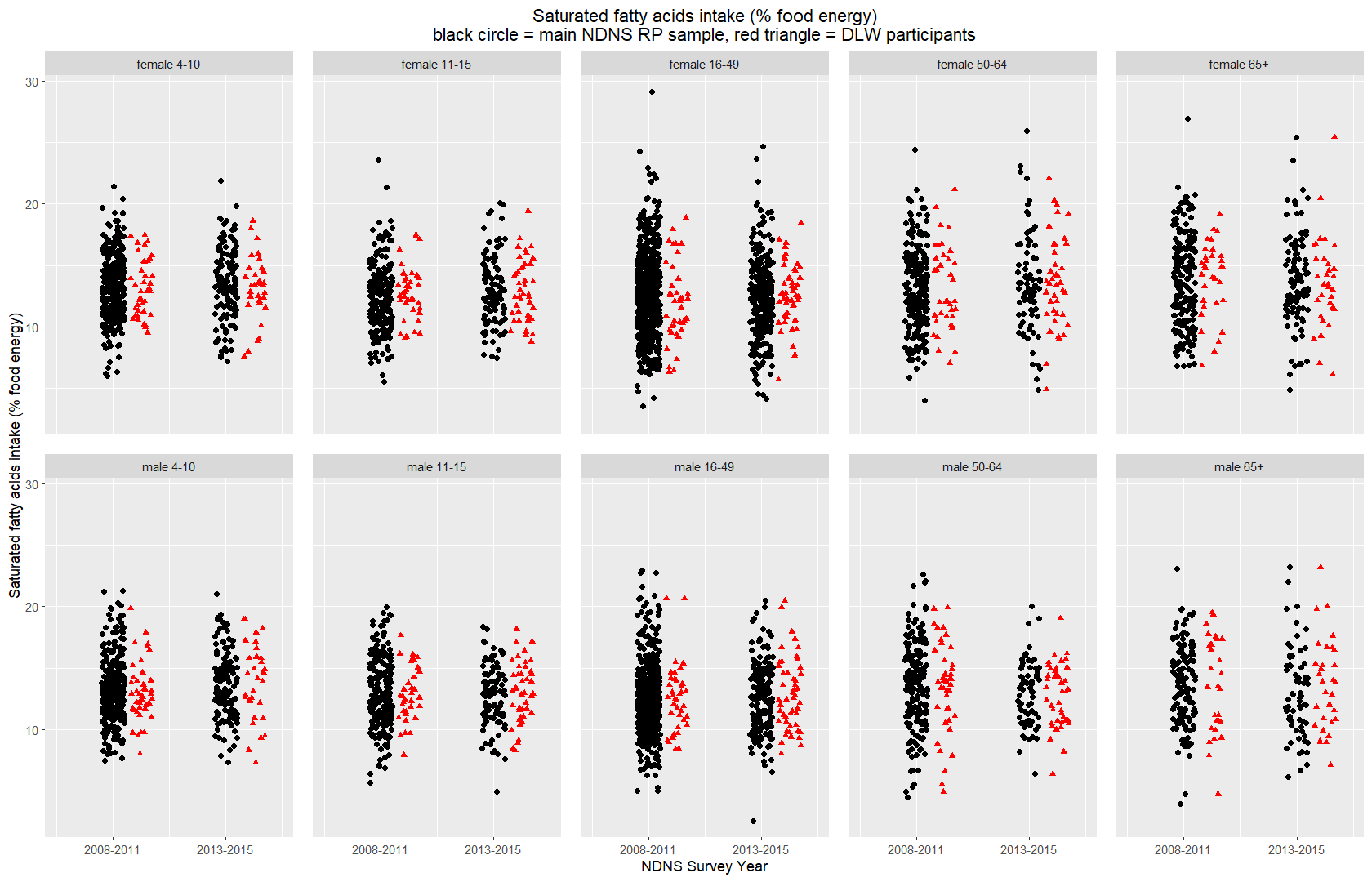

**Supplemental Figure 7:** The comparison of saturated fatty acids intake across all age/sex strata, for doubly labelled water participants *vs.* all participants within the main UK National Diet and Nutrition Survey Rolling Programme, during the measurement periods 2008-2011 and 2013-2015.

Black circles denote the main NDNS RP participants; red triangles denote the doubly labelled water participants.
